# Persistence of Causal Illusions After Extensive Training

**DOI:** 10.3389/fpsyg.2019.00024

**Published:** 2019-01-24

**Authors:** Itxaso Barberia, Miguel A. Vadillo, Javier Rodríguez-Ferreiro

**Affiliations:** ^1^Departament de Cognició, Desenvolupament y Psicologia de la Educació, Universitat de Barcelona, Barcelona, Spain; ^2^Departamento de Psicología Básica, Universidad Autónoma de Madrid, Madrid, Spain; ^3^Institut de Neurociències, Universitat de Barcelona, Barcelona, Spain

**Keywords:** causal illusion, illusion of causality, contingency learning, causal learning, extensive training, Rescorla-Wagner model

## Abstract

We carried out an experiment using a conventional causal learning task but extending the number of learning trials participants were exposed to. Participants in the standard training group were exposed to 48 learning trials before being asked about the potential causal relationship under examination, whereas for participants in the long training group the length of training was extended to 288 trials. In both groups, the event acting as the potential cause had zero correlation with the occurrence of the outcome, but both the outcome density and the cause density were high, therefore providing a breeding ground for the emergence of a causal illusion. In contradiction to the predictions of associative models such the Rescorla-Wagner model, we found moderate evidence against the hypothesis that extending the learning phase alters the causal illusion. However, assessing causal impressions recurrently did weaken participants’ causal illusions.

## Introduction

Several studies have found that, when certain conditions are met, people are easily led to develop erroneous causal beliefs. In the canonical experiment, volunteers are initially informed that their aim is to explore the extent to which a causal relationship exists. For example, they may be asked to find out the extent to which a fictitious drug (a candidate cause) is effective in producing recovery from a fictitious disease (the outcome). Then, participants observe several patients with the disease, presented sequentially on a trial-by-trial basis. For each patient, they are told if the patient took the drug or not (i.e., if the candidate cause is present or not), and whether the patient recovered from the disease or not (if the outcome occurred or not). After seeing all patients, they have to rate the effectiveness of the drug in a numerical scale, usually ranging from 0 to 100. In reality, the experimenter has set up the task for the probability of recovery to be the same both for patients taking the drug and for patients not taking it. Therefore, the normative answer in these conditions should be that the drug is ineffective, because taking it does not increase the chances of recovery. However, under some circumstances, people tend to perceive that the drug is effective, showing what could be called a *causal illusion* or an *illusion of causality* (see Matute et al., 2015, for a review).

Previous research on this subject reveals that two factors play an essential role in the development of causal illusions. In our previous scenario, even if the chances of recovery are the same among patients taking the drug and patients not taking it, the drug has greater chances of being perceived as effective if the recovery is, in general, very frequent (high outcome density) and if there is a high proportion of patients taking the drug (high cause density) (e.g., Alloy and Abramson, 1979; Allan and Jenkins, 1983; Allan et al., 2005; Hannah and Beneteau, 2009; Blanco et al., 2013). Table 1 represents an example with both a high outcome density and a high cause density, illustrating a situation that should encourage strong causal illusions.

**Table 1:** A contingency table showing an example of a situation prone to generating the illusory perception of the drug being effective.

	Patients recovered	Patients not recovered
Patients taking the drug	27 (*a*)	9 (*b*)
Patients not taking the drug	9 (*c*)	3 (*d*)

*Out of 48 patients (i.e., trials) 27 took the drug and recovered from the disease (cell a), 9 took the drug but did not recover (cell b), 9 recovered without taking the drug (cell c), and 3 did not take the drug and did not recover (cell d). Therefore, the probability of recovery is 0.75 both among patients that took the drug [27 out of 36; a/(a+b)] and among patients that did not take the drug [9 out of 12; c/(c+d)]. Also, the outcome density is high (36 out of 48 patients recovered from the disease, irrespective of the intake of the drug), and the cause density is high (36 out of 48 patients took the drug, irrespective of them experiencing subsequent recovery or not). Although the drug does not increase the chances of recovery, a strong causal illusion is expected in this situation.*

The relevance of this phenomenon is partly based on the fact that many erroneous beliefs in our daily life might appear just in the same manner as causal illusions emerge in these simple computer tasks in the lab (Blanco et al., 2015; Matute et al., 2015; Griffiths et al., 2018). This would be the case, for example, for pseudomedicines or “miracle products” with no proven capacity to produce improvement. In these cases, some people develop the conviction of the effectiveness of a treatment, even when there is no real covariation between using the pseudomedicine or the miracle product and the improvement (Matute et al., 2011). In fact, as previously noted by Blanco et al. (2014), pseudomedicines seem to proliferate in contexts that are ideal for the rise of causal illusions: first, they are usually applied to health problems (e.g., back pain, headache) that tend to show spontaneous recovery (high outcome density) and, since they are frequently advertised as lacking side effects, they tend to be used recurrently (high cause density). As far as misbeliefs such as these can strongly impact relevant daily life decisions, understanding the mechanisms by which they are formed, the circumstances in which they most probably proliferate, and the expected evolution of these over time seems essential.

Associative learning models, such as the error-correction algorithm proposed by Rescorla and Wagner (1972) are able to predict the appearance of causal illusions (e.g., Shanks, 1995; Musca et al., 2008; Vadillo et al., 2016; Vadillo and Barberia, 2018). The Rescorla-Wagner model assumes that information about the relationship between different events in the environment is encoded in the form of associations whose strengths are dynamically updated as new evidence is encountered. In a causal learning situation like the one summarized in Table 1, the model assumes that whenever we encounter a patient who takes the drug and recovers from the disease, the link between the mental representations of these two events (drug and recovery) are strengthened, while encountering a patient who takes the drug but does not experience recovery weakens the association between both events. The change in the strength of this association is given by equation:

(1) $$\Delta {\text{V}}_{\text{n}}=\text{α}\cdot \text{β}\cdot (\text{λ}-\sum {\text{V}}_{\text{n}-1})$$

where ΔV_n_ is the change in the associative strength on trial *n*, α, and β are two learning rate parameters dependent on the saliences of the two events to be associated, λ denotes whether the outcome (i.e., the recovery) was observed or not in that trial, and ∑ V_n−1_ is the sum of the associative strengths previously accumulated by all the cues present in that trial. Of note, although the associative strength of a cue (i.e., taking the drug) only changes when that cue is present, the model assumes that in addition to the target cue there is also a constant contextual cue, present in all trials, that competes with the target cue to get associated with the outcome. With this simple implementation, the model predicts that after a representative sequence of trials, the associative strength of the target cue will approach a value close to the objective correlation between taking the drug and recovery (Chapman and Robbins, 1990; Wasserman et al., 1993), as measured by an index called Δ*P* (Allan, 1980), which can be computed as:

(2) ΔP=P(recovery|drug)−P(recovery|∼drug)

In that sense, the Rescorla-Wagner model can be seen as a “rational” theory of causal learning (Shanks, 1995), because once sufficient information has been experienced, the strength of the association between causes and effects will approach the objective degree of correlation between them^1^. However, before reaching the learning asymptote, the model also predicts that the associative strengths will be biased by factors that, from a rational point of view, should play no role at all. Specifically, the model predicts that the development of the associations will be positively biased by the overall probabilities of the two events to be associated (i.e., outcome density and cause density).

**FIGURE 1:**
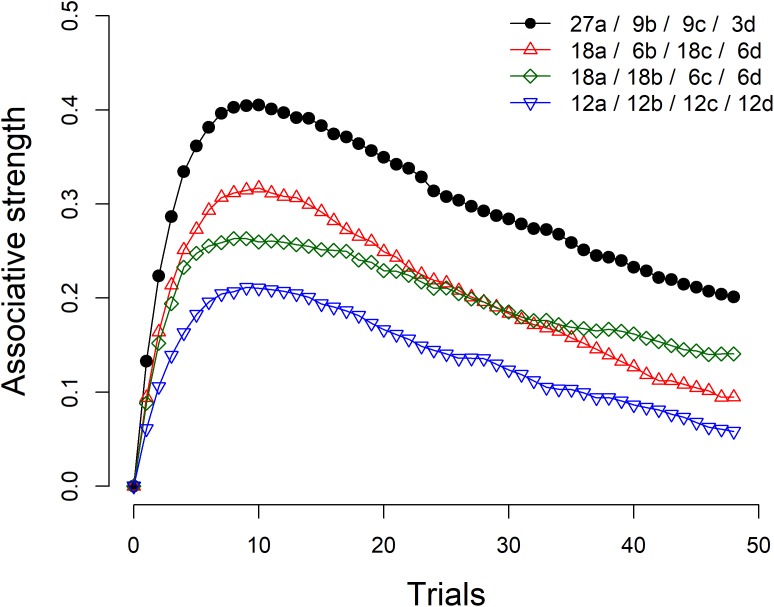
Four simulations of the Rescorla-Wagner model. The figure legend summarizes the number of *a*, *b*, *c*, and *d* trials (see Table 1) included in each simulation. Learning rate parameters α_cue_, α_context_, and β were set to 0.4, 0.2, and 0.6, respectively. The value of λ was set up to 1 for trials in which the outcome was present and to 0 for trials in which the outcome was absent. The figure shows the average results of 2,000 iterations with random trial orders.

As an example, Figure 1 shows the results of four simulations of the Rescorla-Wagner model. The simulation represented by black circles refers to the condition summarized in Table 1. As can be seen, the associative strength between taking the drug and recovering from the disease is strongly biased in the initial trials and slowly declines as more information is gathered. With sufficient training, the associative strength would eventually reach an asymptotic value of zero. The comparison of this condition with the remaining three conditions shows that the size of the initial bias depends heavily on the marginal probabilities of the cause and the effect. The condition denoted by red triangles is identical to the one denoted by black circles, except that in the latter the density of the cause is *P*(drug) = 0.75 while in the former the density of the cause is *P*(drug) = 0.50. Similarly, the condition denoted by green diamonds is identical to the condition denoted by black circles, except that in the latter the density of the effect is *P*(recovery) = 0.75, while in the former the density of the effect is *P*(recovery) = 0.50. Finally, in the last condition, represented by blue inverted triangles, both the density of the cause and the density of the effect are 0.50. The simulations show that increasing any of these probabilities makes a substantial difference in the predictions of the model, even though the degree of relationship between the cause and the effect is zero in all cases.

Determining the extent to which the causal illusion is pre-asymptotic or permanent is crucial, both because of theoretical and applied reasons. At a theoretical level, the question of interest is whether associative models such as Rescorla and Wagner’s can account for the evolution of causal misbeliefs after extended exposure to the candidate cause and the outcome. That is, are these erroneous beliefs subject to normative adjustment following a more extended exposure to them? In applied terms this issue is also relevant as it could help us envisage if misbeliefs in daily life, such as those related to pseudomedicine and miracle products, can be expected to vanish as more information is gathered or if, once implemented, these beliefs remain relatively stable unless other counteractive, e.g., informative or educational, initiatives are introduced. In this sense, it is worth noting that previous evidence in the domain of anti-vaccination and political misinformation suggests that, once erroneous beliefs are accepted as valid, they tend to be quite resistant to any attempt to refute them (e.g., Nyhan and Reifler, 2010; Lewandowsky et al., 2012; Nyhan et al., 2014).

In the present study, we adapted the standard laboratory task used in research on causal illusions to an extended learning situation. Most of the previous experiments exploring causal illusions employed training sessions of about 40 trials in the shortest cases (e.g., Barberia et al., 2013; Blanco et al., 2015; Griffiths et al., 2018) to about 100 trials in the longest cases (e.g., Matute et al., 2011; Yarritu et al., 2014). For this study, we decided to greatly extend the amount of training the participants were exposed to and explore if this manipulation diminished the intensity of the illusion developed.

With this goal in mind, all participants were exposed to a causal learning task typically used in the research on causal illusions (Matute et al., 2015). The scenario involved discovering if a fictitious drug was effective in making patients overcome the crises produced by a fictitious disease. Half of the participants observed 48 patients (Standard Training group or “Standard” group) and then evaluated the effectiveness of the drug, whereas for the other half (Long Training group or “Long” group) the length of training was multiplied by six (48 × 6) and thus, participants in this condition observed a total of 288 patients before evaluating the drug. If causal illusions are a product of incomplete learning, causal ratings given after 48 trials (Standard group) should be higher than those given after 288 trials (Long group).

We note that studies from a related area of research, illusory correlation in stereotype formation (Hamilton and Gifford, 1976), could be relevant for drawing informed hypotheses regarding the results of our present study. Much like in the case of causal illusion experiments, illusory correlation studies rely on contingency detection tasks, in which volunteers tend to overestimate the degree of covariation between two given variables. Importantly, Murphy et al. (2011) demonstrated that the illusory correlation effect might be a preasymptotic or incomplete learning effect. In their experiments, the effect emerged early in training but disappeared after more extended training. However, another paper published that same year (Kutzner et al., 2011) found a persistent illusory correlation effect even after a longer training than that implemented by Murphy et al. (2011). We consider that one important difference between the procedures of both studies was that, whereas Murphy et al. (2011) asked participants to give their evaluations every few trials, Kutzner et al. (2011) only asked for explicit evaluations at the end of training.

This observation led us to explore the effects of recurrent ratings of causal relationship during causal illusion formation as a secondary goal of our research. With this goal in mind, the participants in what we have called the Standard group were, in fact, further trained after observing the first 48 patients and giving their first evaluation of the drug. Specifically, they continued observing patients and evaluating the effectiveness of the drug after every 48 trials, until reaching the same amount of training as those participants in the Long group. Note that this subsequent training does not influence the main measure taken in the Standard group, which happened after the first 48 patients were observed. Inspired by the differences detected between previous results of illusory correlation studies (Kutzner et al., 2011; Murphy et al., 2011), we hypothesized that, whereas no differences would appear between the first causal ratings obtained in the two groups, regardless of training length, repeatedly asking participants to evaluate the effects of the drug might decrease the causal illusion effect.

## Materials and Methods

### Participants

A total of 150 Psychology undergraduate students participated in the experiment in return for course credits, and they were randomly assigned to the Standard group or Long group (75 participants per group, 62 female, mean age = 21.28, *SD* = 7.06 in the Standard group and 63 female, mean age = 19.80, *SD* = 3.26 in the Long group). Sample size was determined following the criteria that participants would be added sequentially until reaching a Bayes factor (BF) higher than 10 in favor or against the alternative hypothesis, i.e., that participants in the Standard group would show a stronger causal illusion than participants in the Long group, as measured by the causal question (see section “Materials and Methods”). If none of these values were reached after 150 participants (75 per group) the experiment would be stopped (see e.g., Wagenmakers et al., 2015, for a similar sampling plan). In the end, the BF did not reach any of the target boundaries and the final sample was of 150 participants. Although this stopping rule was based on a Bayesian approach, we report both Bayesian and frequentist statistics for all analyses. In the few occasions when the results of both types of analyses seem to lead to different conclusions, we highlight these discrepancies. Participants gave written informed consent before participating in the experiment. The study protocol was approved by the ethics committee of the University of Barcelona (Institutional Review Board IRB00003099).

### Procedure

The experiment was programmed using Xojo^2^ and all the instructions were provided in Spanish. Participants were exposed to a standard causal learning task. Specifically, they were asked to imagine that they were specialists in a strange and dangerous disease called Lindsay Syndrome (e.g., Blanco et al., 2011, 2013; Matute et al., 2011). They were further told that the crises produced by this disease might be overcome with a new experimental drug (Batatrim) whose effectiveness was still to be determined. In order to evaluate the effectiveness of this drug, participants were told that they would see the medical records of several patients, presented one by one. Each patient would be suffering from a crisis and some of them would receive the drug whereas others would not receive anything. Whether the patient had taken the drug or not, participants would have to predict if the patient would overcome the crisis. The prediction would be followed by a feedback indicating if the patient overcame the crisis or not. Participants were further informed that, since they would see many patients, they were free to take short rests whenever they needed. (They were informed that they were not allowed to perform any other activity, such as using their cellphones, during these periods.) The instructions ended informing the participants that their goal was to find out if the drug was effective and that, after observing a good number of patients, they would be asked some questions.

After the initial instructions, participants were exposed to a sequence of 288 patients, presented one after the other. On each trial, the same suffering emoticon appeared in the left side of the screen, together with an image of a pill on the right side of the screen, if the trial corresponded to a patient that had taken the drug, or with the same pill crossed out, if the trial corresponded to a patient that did not take the drug. A text in the upper side of the screen indicated “This patient suffers from a crisis and receives Batatrim” or “This patient suffers from a crisis and does not receive anything,” respectively. An additional text in the lower part of the screen asked participants “Do you think the patient would overcome the crisis?” Participants could answer by clicking on one of two (yes/no) buttons. After the participants had made their prediction, the image of the suffering emoticon and all the texts disappeared from the screen for 1 s. After this period, the emoticon reappeared on the screen, either with the same suffering expression or with a happy expression, and with the “The patient did not overcome the crisis” or “The patient overcame the crisis!” text, respectively. One second later, an additional button that allowed participants to see the next patient was activated.

We assessed the causal illusion by means of three questions: the causal question and two conditional probability questions. For the causal question, which was our main dependent variable (see e.g., Blanco et al., 2011; Barberia et al., 2013, for similar questions), participants were asked “To what extent do you think that Batatrim is effective to overcome the crises produced by the Lindsay Syndrome?” and they could answer in a scale from 0 (not effective at all) to 100 (totally effective). Given that the normative calculation of covariation between cause and outcome depends on the direct comparison between cause-present and cause-absent outcome probabilities, we also introduced two conditional probability questions in order to gain insight on the process that might underlie the participants’ responses to the causal question. For these two conditional probability questions, participants were asked to estimate the number of patients that would overcome the crisis among 100 new patients taking the drug [*P*(recovery|drug) question], and among 100 new patients not taking the drug [*P*(recovery|∼drug) question]: “Imagine 100 NEW PATIENTS that are suffering a crisis produced by the Lindsay Syndrome and TAKE BATATRIM [DO NOT TAKE ANYTHING]. How many do you think they will overcome the crisis?” The order in which the questions were answered was balanced across participants: about half of the participants of each condition answered the causal question first and then the two questions referring to the conditional probabilities (also balanced in order), whereas for the other half this order was reversed, resulting in four possible orders.

In the case of the participants in the Standard group, after each block of 48 patients, participants answered the causal question and conditional probability questions and they were told that they would continue observing more patients. They were requested to answer the three questions (causal question and conditional probability questions) again after every 48 trials and, therefore, they experienced a total of six blocks of ratings until reaching the same amount of training as the participants in the Long group. For the Long group these questions were answered only after all 288 patients had been observed.

Participants in both groups were exposed to six blocks of randomly presented 48 trials, of which 27 corresponded to *a* trials, 9 to *b* trials, 9 to *c* trials, and 3 to *d* trials (see Table 1). Every 48 trials the program would re-randomize the sequence. For the Standard group, this re-randomization coincided with the moment in which the causal and conditional probability questions were answered after each block of trials. Note that the situation was set up so that the contingency between the drug and recovery was zero, because the probability of recovery among patients that took the drug [i.e., *P*(recovery|drug) = *a*/(*a*+*b*) = 27/(27+9) = 0.75] was the same as the probability of recovery among those patient that did not take any drug [i.e., *P*(recovery|∼drug) = *c*/(*c*+*d*) = 9/(9+3) = 0.75]. Moreover, both the outcome density [*P*(recovery) = (*a*+*c*)/(*a*+*b*+*c*+*d*) = (27+9)/(27+9+9+3) = 0.75] and the cause density [*P*(drug) = (*a*+*b*)/(*a*+*b*+*c*+*d*) = (27+9)/(27+9+9+3) = 0.75] were high, therefore emulating the conditions under which the causal illusions are known to reach strong levels (Blanco et al., 2013; Matute et al., 2015, see simulations in Figure 1).

## Results

The dataset for this study can be found at: https://osf.io/35ytz/

**FIGURE 2:**
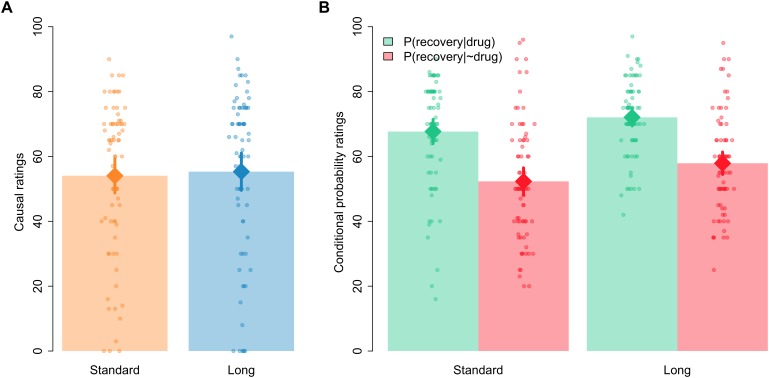
Mean causal ratings **(A)** and conditional probability ratings **(B)** after 48 trials in the Standard group and after all 288 trials in the Long group. Error bars denote 95% confidence intervals.

All the analyses reported in this section were conducted with JASP, https://jasp-stats.org/ (JASP Team, 2018; see also Wagenmakers et al., 2018). Following Wagenmakers et al. (2018, see their Table 1) we interpret BFs between 1 and 3 as anecdotal evidence, BFs between 3 and 10 as moderate evidence, and BFs >10 as strong-to-extreme evidence. Figure 2 shows the first causal and conditional probabilities’ ratings given by participants of each group, that is, the ratings given after 48 trials in the Standard group and the ratings given after all 288 trials for the Long group. As can be seen, mean causal ratings (our main dependent variable, see panel A) were similar in both groups. A Bayesian *t*-test contrasting the null hypothesis against the alternative hypothesis that ratings would be different in both groups (modeled as a two-sided Cauchy distribution with *r* = 0.707) yielded a BF_01_ = 5.43, suggesting moderate evidence in favor of the null hypothesis. Frequentist statistics yielded no significant differences between both groups, *t*(148) = −0.317, *p* = 0.752, *d* = −0.052.

In the case of the estimated conditional probabilities, visual inspection of Figure 2 (panel B) indicates that the probability of recovery was assumed to be higher among those patients who took the drug than among those patients who did not take the drug, which would be consistent with participants giving causal ratings higher than zero in the causal question. Also, Figure 2 suggests that the ratings of both probabilities seemed to be somewhat higher in the Long than in the Standard group. A 2 [question: *P*(recovery|drug) vs. *P*(recovery|∼drug)] × 2 [group: Standard vs. Long] Bayesian ANOVA confirmed these impressions. The model including only the main effects of question and group outperformed all other models, with a BF = 2.70 over the model including just question, a BF = 5.58 over the model including both main effects and their interaction, and a BF = 1.99 × 10^13^ over the model including just the main effect of group. The frequentist ANOVA yielded significant main effects of question and group, *F*(1,148) = 68.690, *p* < 0.001, η_p_^2^ = 0.317, and *F*(1,148) = 7.266, *p* = 0.008, η_p_^2^ = 0.047, respectively, but the interaction failed to reach significance, *F* < 1.

A potential criticism to these results raised by a reviewer is that perhaps participants had already reached the learning asymptote after just 48 trials, in which case associative models of learning predict no further change with additional training. (Note, however, that the asymptotic level predicted by the Rescorla-Wagner model is an associative strength of zero.) To explore this possibility, we analyzed the trial-by-trial predictions made by participants. Specifically, we calculated the proportion of outcome predictions (i.e., “yes” responses) in cause-present and cause-absent trials for the initial 48 trials in the Standard group and for the last 48 trials in the Long group. The descriptive statistics show that outcome predictions tended to be higher for the Long group, both in cause-present (Standard: Mean = 0.82, *SD* = 0.12; Long: Mean = 0.86, *SD* = 0.12) and cause-absent trials (Standard: Mean = 0.50, *SD* = 0.23; Long: Mean = 0.73, *SD* = 0.22). These impressions were confirmed by a 2 [type of trial: cause-present vs. cause-absent] × 2 [group: Standard vs. Long] Bayesian ANOVA. The model including both main effects and their interaction achieved the best performance, with a BF = 9317.55 over the model including just the two main effects and BFs > 4.11 × 10^9^ over the rest of models. The frequentist ANOVA yielded significant main effects of type of trial, *F*(1,148) = 113.39, *p* < 0.001, η_p_^2^ = 0.434, and group, *F*(1,148) = 42.47, *p* < 0.001, η_p_^2^ = 0.223, and a significant type of trial × group interaction, *F*(1,148) = 21.01, *p* < 0.001, η_p_^2^ = 0.124. The analysis of the interaction showed that outcome predictions significantly differed between groups in the case of the cause-absent trials, *t*(148) = −6.255, *p* < 0.001, *d* = −1.021, BF_10_ = 2.39 × 10^6^, and only marginally differed between groups for the cause-present trials, *t*(148) = −1.862, *p* = 0.065, *d* = −0.304, BF_01_ = 1.17.

**FIGURE 3:**
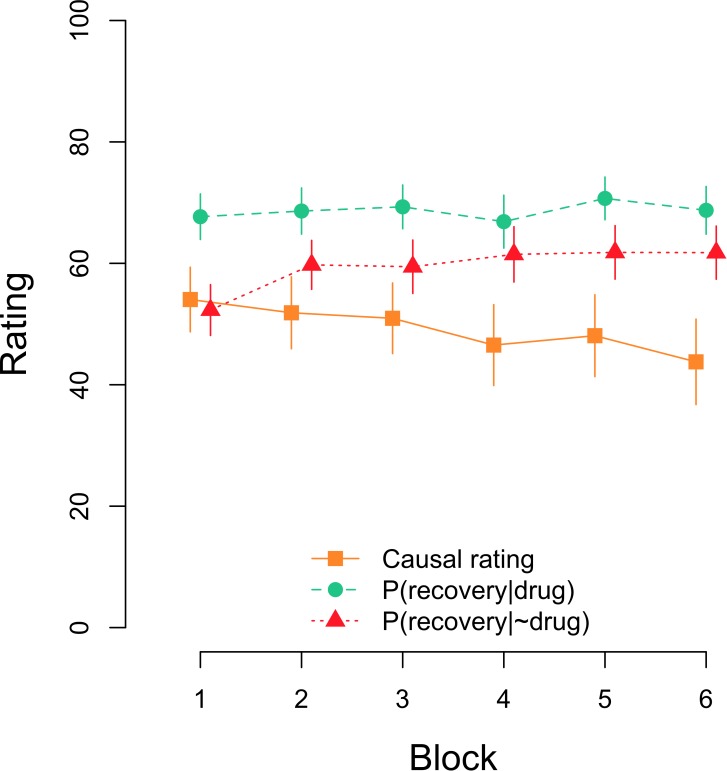
Mean causal and conditional probability ratings after each block of 48 trials in the Standard group. Error bars denote 95% confidence intervals.

As noted in the introduction, a complementary goal of our study was to explore if the fact that the participants were requested to answer the causal and conditional probability questions several times during training would affect their estimations. Figure 3 shows the evolution of causal and conditional probabilities’ estimations over time in the Standard group. A Bayesian repeated measures ANOVA on causal ratings yielded a BF_10_ = 3.91 in favor of the model including the factor block over the null model. This result was confirmed by an equivalent frequentist ANOVA, *F*(5,370) = 3.641, *p* = 0.003, η_p_^2^ = 0.047. A direct (two-sided) comparison of blocks 1 and 6 shows that causal judgments declined from the first to the last block of testing, *t*(74) = 2.720, *p* = 0.008, *d* = 0.314, BF_10_ = 3.84. Regarding estimated conditional probabilities, Figure 3 suggests that there was an increase over time in the estimation of the *P*(recovery|∼drug) but not in the estimation of the *P*(recovery|drug). A Bayesian repeated measures ANOVA with the factors question [i.e., *P*(recovery|drug) vs. *P*(recovery|∼drug)] and block (1–6) showed that the model including only question outperformed the rest of models, with a BF = 2.34 over the model including question and block, a BF = 19.3 over the model including both main effects and their interaction, and a BF = 3.60 × 10^16^ over the model including just block. Interestingly, the frequentist ANOVA returned significant effects not only for question, *F*(1,74) = 21.372, *p* < 0.001, η_p_^2^ = 0.224, but also for block, *F*(5,370) = 3.948, *p* = 0.002, η_p_^2^ = 0.051, and for the interaction as well, *F*(5,370) = 3.047, *p* = 0.010, η_p_^2^ = 0.040. Further separate ANOVAs for each of the questions revealed no effect of block in the case of the *P*(recovery|drug), BF_01_ = 72.63, *F* < 1, but a significant block effect for the *P*(recovery|∼drug), BF_10_ = 355.50, *F*(5,370) = 5.826, *p* < 0.001, η_p_^2^ = 0.073.

Another way of exploring the effect of repeated testing is to compare ratings at the end of the experiment in both experimental conditions. At that time, both groups of participants have experienced the same number of trials, but participants in the Standard group have already been tested five times. The descriptive statistics show that at that time causal ratings in the Standard group were numerically lower (Mean = 43.77, *SD* = 31.08) than causal ratings in the Long group (Mean = 55.31, *SD* = 25.46). The Bayesian analysis yielded a BF_10_ = 2.905 in favor of the hypothesis that causal ratings were different in both groups over the null hypothesis. A frequentist *t*-test confirmed that the comparison was statistically significant, *t*(148) = −2.486, *p* = 0.014, *d* = −0.406. A similar comparison of conditional probability ratings revealed that, overall, estimations of *P*(recovery|drug) tended to be higher (Standard: Mean = 68.73, *SD* = 17.36; Long: Mean = 72.07, *SD* = 11.81) than estimations of *P*(recovery|∼drug) (Standard: Mean = 61.76, *SD* = 19.32; Long: Mean = 57.91, *SD* = 15.33). These ratings were analyzed through a 2 [question: *P*(recovery|drug) vs. *P*(recovery|∼drug)] × 2 [group: Standard vs. Long] Bayesian ANOVA. The model including only question outperformed all other models, with a BF = 3.18 over the model including both main effects and their interaction, a BF = 5.89 over the model including both main effects, and a BF = 1.29 × 10^8^ over the model including just group. Note, however, that the frequentist ANOVA yielded both a significant main effect of question, *F*(1,148) = 44.038, *p* < 0.001, η_p_^2^ = 0.229, and a significant question × group interaction, *F*(1,148) = 5.093, *p* = 0.025, η_p_^2^ = 0.033, but not a main effect of group, *F* < 1. The analysis of this interaction showed that estimations of *P*(recovery|drug) and *P*(recovery|∼drug) differed both in the Standard group, *t*(74) = 2.716, *p* = 0.008, *d* = 0.314, BF_10_ = 3.80, and in the Long group, *t*(74) = 7.518, *p* < 0.001, *d* = 0.868, BF_10_ = 9.22 × 10^7^.

We also conducted a parallel set of frequentist analyses including the order in which the causal question and conditional probability estimations were answered by the participants. These new analyses showed that the order of presentation of the questions did not significantly impact our dependent variables (see Supplementary Materials).

## Discussion

The main goal of the present study was to investigate the course of causal illusions after an extensive training with the candidate cause and its potential connection with the outcome. To reach this aim, we compared causal impressions originated after a conventional length of training (48 trials, Standard group) with those emerged after a markedly extended training (288 trials, Long group). Our results indicated that lengthening the amount of training did not produce a significant decrease in the intensity of causal illusions. Specifically, there was no change neither in the causal ratings given by the participants nor in the difference in their estimation of the two conditional probabilities.

This result is at odds with associative models such as Rescorla-Wagner model, which predict that biased causal impressions at zero contingency conditions would be pre-asymptotic and should therefore adjust to the normative absence of contingency as training moves forward. However, other models could accommodate the present results, such as some rule-based models. These models postulate that people encode the frequencies of the different events (cells *a*, *b*, *c*, and *d* in Table 1) or conditional probabilities of the outcome in the presence and absence of the candidate cause (see Equation 2) and apply some algebraic combination of them to form their causal impressions (see Perales and Shanks, 2007; Perales et al., 2017, for reviews). For example, the *weighted Δ*P* model* (e.g., Lober and Shanks, 2000) assumes that people compute the two conditional probabilities involved in the calculation of Δ*P* but assign different weights to the probability of the outcome given the potential cause [*P*(recovery|drug) in Equation 2] and to the probability of the outcome when the potential cause is absent [*P*(recovery|∼drug) in Equation 2]. Typically, the best fit is found if the probability of the outcome is given more weight when the potential cause is present than when it is absent (see Lober and Shanks, 2000; Perales and Shanks, 2007). As another example, the *Evidence Integration (EI) rule* (Perales and Shanks, 2007) proposes that causal ratings result from the comparison between confirmatory (cells *a* and *d* in Table 1) and disconfirmatory (cells *b* and *c* in Table 1) information, and allows the four cells of the contingency table to be given different weights (see Perales and Shanks, 2007, p. 583, for more details about the proposed rule). Perales and Shanks (2007) found in their meta-analysis that the best fit was for weights (*w*) ordering corresponding to *w_a_* > *w_b_* > *w_c_* > *w_d_*. Interestingly, both of these alternative models would anticipate a biased (non-zero) causal impression for the participants in our experiment, if the typical weighting is applied to the conditional probabilities or cell frequencies. Crucially, as long as the two relevant conditional probabilities (*weighted* Δ*P model*) or the relative frequencies of the four cells in the contingency table (*EI rule*) remain unaltered along training, the predictions of these models would be equal for any amount of trials, which means that these models do not anticipate changes in mean causal impressions after 48 trials versus 288 trials in our experiment.

Regarding previous results on causal illusions literature pointing out in the same direction as the present study, Blanco et al. (2011) carried out two studies using a causal learning task with a cover story similar to the one employed in our study. Their Experiment 1 involved 50 trials and their Experiment 2 was composed of 100 trials. When they performed a cross-experiments comparison they found that, if anything, causal illusions were stronger in their second experiment. However, their procedure involved an active contingency learning task, i.e., participants could decide in every trial if they wanted the candidate cause to be present or not, that is, if they wanted to administer the drug or not. Therefore, participants could decide the cause density to which they were exposed. Since, as discussed in the Introduction, it has been previously shown that higher cause density produces stronger causal illusions, and Blanco et al. (2011) participants tended to (at least marginally) increase the cause density they were exposed to over blocks of trials, this cause density could have affected the evolution of causal impressions over time. To this respect, the present experiment shows a sustained causal illusion in a passive task in which the cause density is externally controlled by the experimenter and kept constant over all training.

This finding has practical implications, because it suggests that causal misbeliefs, once acquired, might be quite resistant to change, even in light of persistent covariation information that contradicts such beliefs. As we have already noted, causal illusions are widely present in our everyday life, and they influence some relevant quotidian decisions like those related to health. Pseudomedicines tend to be repeatedly applied to health conditions associated to high spontaneous recovery rates (Blanco et al., 2014). These high cause density and high outcome density situations are ideal for the appearance of causal illusions. According to our results, we cannot expect the perceived effectiveness of these pseudomedicines or miracle products to spontaneously diminish, even after extended zero-contingency experiences with them. This finding points out the necessity to develop and apply specific interventions (Barberia et al., 2013, 2018) aimed to prevent the appearance of causal illusions or to reduce their influence. As mentioned in the Introduction, this result dovetails with previous research showing that, once accepted as valid, erroneous ideas about topics as diverse as health or politics tend to be resistant to change (e.g., Nyhan and Reifler, 2010; Lewandowsky et al., 2012; Nyhan et al., 2014).

Superficially, our results might seem contradictory with those of previous studies where causal ratings (López et al., 1999; see also Shanks, 1995) or illusory correlations (Murphy et al., 2011) did decline over training. It is worth noting that we too found a decrease in causal illusions, but only after repeated testing. Taken together, our results and previous evidence suggest that the decline might be driven by repeated testing and not extensive training. This result implies that the fact of asking participants to repeatedly evaluate the potential cause may make a difference in the way they solve the task. We hypothesize that, by asking participants to estimate both the *P*(recovery|drug) and *P*(recovery|∼drug) in our experiment, we could have brought their attention to the comparison of these two conditional probabilities when evaluating the effectiveness of the drug. In fact, previous debiasing interventions that have proven effective in decreasing causal illusions (Barberia et al., 2013) have focused on instructing participants on the importance of considering not only the *P*(recovery|drug) or the drug-recovery coincidences, but also the *P*(recovery|∼drug), which might be typically given less importance. Note that this idea might be implemented on the previously discussed rule-based models as changes in the weights given to the different pieces of evidence. It could also be the case that participants start the task with some prior assumptions about both the *P*(recovery|drug) and *P*(recovery|∼drug), expecting the first to be high and the second to be low. This could be a consequence of the specific cover story employed in this experiment, involving a drug and the recovery from a disease. Alternatively, it could be associated to a more general default tendency to *a priori* assume that the cause under examination produces the outcome, and to ignore the potential influence of other unknown generative causes on the same outcome. When participants face the task, the prior about the *P*(recovery|drug) would reasonably fit the actual data they encounter (a lot of *a* trials and much fewer *b* trials), whereas the *P*(recovery|∼drug) would show a stronger discrepancy with participants’ prior. This might require special attention to be driven to trials in which recovery happens without the drug in order to this prior to be modified and recurrently asking participant to estimate the expected percentage of recovery among patients not taking the drug might precisely act in this direction. It is also a possibility that, by asking participants to estimate these two conditional probabilities, we untangled the inherent ambiguity of the causal question (Buehner et al., 2003; Baker et al., 2005), making participants focus on those probabilities when evaluating the effectiveness of the drug, as predicted by the Δ*P* model.

To conclude, the present study suggests that causal illusions are not the consequence of incomplete learning, because they show resistance to extensive training. This result implies that erroneous causal beliefs of daily life are not expected to disappear just because persistent empirical evidence against them is gathered. Moreover, our analyses indicate that, the fact of recurrently asking people to evaluate the potential causal relationship and to estimate the conditional probabilities involved in the contingency calculation, decreases the intensity of the illusion. Further studies should investigate how exactly the introduction of recurrent ratings during contingency detection tasks could be applied to real-life situations in order to positively influence participants’ decisions regarding the existence of causal relationships.

## Author Contributions

IB, MV, and JR-F contributed to the conception and design of the study. IB programmed the computer task. MV performed the statistical analysis. IB and MV wrote the first draft of the manuscript. All authors contributed to manuscript revision, read, and approved the submitted version.

## Conflict of Interest Statement

The authors declare that the research was conducted in the absence of any commercial or financial relationships that could be construed as a potential conflict of interest.

## Footnotes

1 It is worth noting that, for the asymptotic predictions of the Rescorla-Wagner model to converge with Δ*P*, it is necessary to assume a “restricted” version of the model, where β is assumed to have the same value both in trials in which the outcome occurs and in trials in which it does not occur. An “unrestricted” version of the model, allowing β to vary depending on the presence or absence of the outcome, is able to predict deviations from Δ*P* on causal ratings at asymptote (e.g., Wasserman et al., 1993; Buehner and Cheng, 1997; Lober and Shanks, 2000; Buehner et al., 2003). However, this is only the case for non-zero (positive and negative) correlation values, whereas unrestricting the model does not alter the predictions for situations in which there is an absence of contingency (i.e., Δ*P* = 0), as it is the case in settings exploring causal illusions.

2 https://www.xojo.com/
