# Supplementary material for: Persistence of Causal Illusions After Extensive Training
Source: Front Psychol. 2019 Jan 24;10:24. doi: 10.3389/fpsyg.2019.00024 (PMC6353834; doi:10.3389/fpsyg.2019.00024)
Supplement: Supplementary file 1 [file Table_1.DOCX]

**Supplementary material: Analyses including Order**

Variables:

- Condition: Standard vs. Long groups
- Order: the four possible orders in which the causal question and conditional probability estimations were presented
- Block: each of the testing blocks (only in Standard group)
- Question: P(recovery│drug) vs. P(recovery│~drug)
- Trial: cause-present vs. cause-absent trials

**1 First causal rating in Standard vs. Long**

| **ANOVA - CausalJudgment1** | | | | | | | | | | | |
| --- | --- | --- | --- | --- | --- | --- | --- | --- | --- | --- | --- |
| **Cases** | | **Sum of Squares** | | **df** | | **Mean Square** | | **F** | | **p** | |
| Condition |  | 56.32 |  | 1 |  | 56.32 |  | 0.092 |  | 0.762 |  |
| Order |  | 1471.10 |  | 3 |  | 490.37 |  | 0.802 |  | 0.495 |  |
| Condition x Order |  | 451.87 |  | 3 |  | 150.62 |  | 0.246 |  | 0.864 |  |
| Residual |  | 86812.83 |  | 142 |  | 611.36 |  |  |  |  |  |
|  | | | | | | | | | | | |
| *Note.*  Type III Sum of Squares | | | | | | | | | | | |

**2 First estimations of conditional probabilities in Standard vs. Long**

| **Within Subjects Effects** | | | | | | | | | | | | | | | | |
| --- | --- | --- | --- | --- | --- | --- | --- | --- | --- | --- | --- | --- | --- | --- | --- | --- |
|  | | | **Sum of Squares** | | | | **df** | | **Mean Square** | | | | **F** | | **p** | |
| Question | |  | 16467.85 | | |  | 1 |  | 16467.85 | | |  | 69.589 |  | < .001 |  |
| Question x Condition | |  | 31.76 | | |  | 1 |  | 31.76 | | |  | 0.134 |  | 0.715 |  |
| Question x Order | |  | 1074.59 | | |  | 3 |  | 358.20 | | |  | 1.514 |  | 0.214 |  |
| Question x Condition x Order | |  | 567.23 | | |  | 3 |  | 189.08 | | |  | 0.799 |  | 0.496 |  |
| Residual | |  | 33603.74 | | |  | 142 |  | 236.65 | | |  |  |  |  |  |
|  | | | | | | | | | | | | | | | | |
| *Note.*  Type III Sum of Squares | | | | | | | | | | | | | | | | |
| **Between Subjects Effects** | | | | | | | | | | | | | |  |  |  |
|  | | **Sum of Squares** | | **df** | | **Mean Square** | | | | **F** | | **p** | |  |  |  |
| Condition |  | 1884 |  | 1 |  | 1883.6 | | |  | 7.654 |  | 0.006 |  |  |  |  |
| Order |  | 1191 |  | 3 |  | 396.9 | | |  | 1.613 |  | 0.189 |  |  |  |  |
| Condition x Order |  | 1923 |  | 3 |  | 640.9 | | |  | 2.604 |  | 0.054 |  |  |  |  |
| Residual |  | 34947 |  | 142 |  | 246.1 | | |  |  |  |  |  |  |  |  |
|  | | | | | | | | | | | | | |  |  |  |
| *Note.*  Type III Sum of Squares | | | | | | | | | | | | | |  |  |  |

**3 Trial-by-trial predictions in Standard vs. Long**

| **Within Subjects Effects** | | | | | | | | | | | | | | | | |
| --- | --- | --- | --- | --- | --- | --- | --- | --- | --- | --- | --- | --- | --- | --- | --- | --- |
|  | | | **Sum of Squares** | | | | **df** | | **Mean Square** | | **F** | | | | **p** | |
| Trial | |  | 3.847 | | |  | 1 |  | 3.847 |  | 111.239 | | |  | < .001 |  |
| Trial x Condition | |  | 0.715 | | |  | 1 |  | 0.715 |  | 20.684 | | |  | < .001 |  |
| Trial x Order | |  | 0.074 | | |  | 3 |  | 0.025 |  | 0.715 | | |  | 0.545 |  |
| Trial x Condition x Order | |  | 0.027 | | |  | 3 |  | 0.009 |  | 0.257 | | |  | 0.857 |  |
| Residual | |  | 4.911 | | |  | 142 |  | 0.035 |  |  | | |  |  |  |
|  | | | | | | | | | | | | | | | | |
| *Note.*  Type III Sum of Squares | | | | | | | | | | | | | | | | |
| **Between Subjects Effects** | | | | | | | | | | | | | |  |  |  |
|  | | **Sum of Squares** | | **df** | | **Mean Square** | | | | **F** | | **p** | |  |  |  |
| Condition |  | 1.344 |  | 1 |  | 1.344 | | |  | 42.108 |  | < .001 |  |  |  |  |
| Order |  | 0.087 |  | 3 |  | 0.029 | | |  | 0.911 |  | 0.437 |  |  |  |  |
| Condition x Order |  | 0.051 |  | 3 |  | 0.017 | | |  | 0.531 |  | 0.662 |  |  |  |  |
| Residual |  | 4.533 |  | 142 |  | 0.032 | | |  |  |  |  |  |  |  |  |
|  | | | | | | | | | | | | | |  |  |  |
| *Note.*  Type III Sum of Squares | | | | | | | | | | | | | |  |  |  |

**4 Repeated causal ratings in Standard**

| **Within Subjects Effects** | | | | | | | | | | | | | | | | | | |
| --- | --- | --- | --- | --- | --- | --- | --- | --- | --- | --- | --- | --- | --- | --- | --- | --- | --- | --- |
|  | | | **Sum of Squares** | | | | | | | **df** | | | **Mean Square** | | **F** | | **p** | |
| Block | |  | 5282 | | | | ᵃ | | | 5 | | ᵃ | 1056.4 | ᵃ | 3.610 | ᵃ | 0.003 | ᵃ |
| Block x Order | |  | 4859 | | | | ᵃ | | | 15 | | ᵃ | 323.9 | ᵃ | 1.107 | ᵃ | 0.348 | ᵃ |
| Residual | |  | 103885 | | | |  | | | 355 | |  | 292.6 |  |  |  |  |  |
|  | | | | | | | | | | | | | | | | | | |
| *Note.*  Type III Sum of Squares | | | | | | | | | | | | | | | | | | |
| ᵃ Mauchly's test of sphericity indicates that the assumption of sphericity is violated (p < .05). | | | | | | | | | | | | | | | | | | |
| **Between Subjects Effects** | | | | | | | | | | | | | |  |  |  |  |  |
|  | | **Sum of Squares** | | **df** | | **Mean Square** | | | **F** | | | **p** | |  |  |  |  |  |
| Order |  | 17670 |  | 3 |  | 5890 | |  | 1.946 | |  | 0.130 |  |  |  |  |  |  |
| Residual |  | 214918 |  | 71 |  | 3027 | |  |  | |  |  |  |  |  |  |  |  |
|  | | | | | | | | | | | | | |  |  |  |  |  |
| *Note.*  Type III Sum of Squares | | | | | | | | | | | | | |  |  |  |  |  |

**5 Direct comparison between blocks 1 and 6 in Standard**

| **Within Subjects Effects** | | | | | | | | | | | | | | |
| --- | --- | --- | --- | --- | --- | --- | --- | --- | --- | --- | --- | --- | --- | --- |
|  | | | **Sum of Squares** | | | | **df** | | **Mean Square** | | **F** | | **p** | |
| Block | |  | 3924.2 | | |  | 1 |  | 3924.2 |  | 7.214 |  | 0.009 |  |
| Block x Order | |  | 909.3 | | |  | 3 |  | 303.1 |  | 0.557 |  | 0.645 |  |
| Residual | |  | 38621.0 | | |  | 71 |  | 544.0 |  |  |  |  |  |
|  | | | | | | | | | | | | | | |
| *Note.*  Type III Sum of Squares | | | | | | | | | | | | | | |
| **Between Subjects Effects** | | | | | | | | | | | | | |  |
|  | | **Sum of Squares** | | **df** | | **Mean Square** | | | | **F** | | **p** | |  |
| Order |  | 4151 |  | 3 |  | 1383.8 | | |  | 1.432 |  | 0.241 |  |  |
| Residual |  | 68594 |  | 71 |  | 966.1 | | |  |  |  |  |  |  |
|  | | | | | | | | | | | | | |  |
| *Note.*  Type III Sum of Squares | | | | | | | | | | | | | |  |

**6 Repeated estimations of conditional probabilities in Standard**

| **Within Subjects Effects** | | | | | | | | | | | | | | | | |
| --- | --- | --- | --- | --- | --- | --- | --- | --- | --- | --- | --- | --- | --- | --- | --- | --- |
|  | | | | | | | **Sum of Squares** | | **df** | | **Mean Square** | | **F** | | **p** | |
| Question | | | | | |  | 19525 |  | 1 |  | 19524.9 |  | 22.069 |  | < .001 |  |
| Question x Order | | | | | |  | 3508 |  | 3 |  | 1169.2 |  | 1.322 |  | 0.274 |  |
| Residual | | | | | |  | 62816 |  | 71 |  | 884.7 |  |  |  |  |  |
| Block | | | | | |  | 3422 | ᵃ | 5 | ᵃ | 684.5 | ᵃ | 3.958 | ᵃ | 0.002 | ᵃ |
| Block x Order | | | | | |  | 2851 | ᵃ | 15 | ᵃ | 190.1 | ᵃ | 1.099 | ᵃ | 0.355 | ᵃ |
| Residual | | | | | |  | 61392 |  | 355 |  | 172.9 |  |  |  |  |  |
| Question x Block | | | | | |  | 2199 | ᵃ | 5 | ᵃ | 439.8 | ᵃ | 3.116 | ᵃ | 0.009 | ᵃ |
| Question x Block x Order | | | | | |  | 2960 | ᵃ | 15 | ᵃ | 197.3 | ᵃ | 1.398 | ᵃ | 0.145 | ᵃ |
| Residual | | | | | |  | 50101 |  | 355 |  | 141.1 |  |  |  |  |  |
|  | | | | | | | | | | | | | | | | |
| *Note.*  Type III Sum of Squares | | | | | | | | | | | | | | | | |
| ᵃ Mauchly's test of sphericity indicates that the assumption of sphericity is violated (p < .05). | | | | | | | | | | | | | | | | |
| **Between Subjects Effects** | | | | | | | | | | | |  |  |  |  |  |
|  | | **Sum of Squares** | | **df** | | **Mean Square** | | **F** | | **p** | |  |  |  |  |  |
| Order |  | 3621 |  | 3 |  | 1207 |  | 0.836 |  | 0.479 |  |  |  |  |  |  |
| Residual |  | 102510 |  | 71 |  | 1444 |  |  |  |  |  |  |  |  |  |  |
|  | | | | | | | | | | | |  |  |  |  |  |
| *Note.*  Type III Sum of Squares | | | | | | | | | | | |  |  |  |  |  |

**7 Last causal judgement in Standard vs. Long**

| **ANOVA - CausalJudgment6** | | | | | | | | | | | |
| --- | --- | --- | --- | --- | --- | --- | --- | --- | --- | --- | --- |
| **Cases** | | **Sum of Squares** | | **df** | | **Mean Square** | | **F** | | **p** | |
| Condition |  | 4921 |  | 1 |  | 4920.8 |  | 6.045 |  | 0.015 |  |
| Order |  | 2554 |  | 3 |  | 851.2 |  | 1.046 |  | 0.374 |  |
| Condition x Order |  | 1278 |  | 3 |  | 426.0 |  | 0.523 |  | 0.667 |  |
| Residual |  | 115598 |  | 142 |  | 814.1 |  |  |  |  |  |
|  | | | | | | | | | | | |
| *Note.*  Type III Sum of Squares | | | | | | | | | | | |

**8 Last estimations of conditional probabilities in Standard vs. Long**

| **Within Subjects Effects** | | | | | | | | | | | | | | | | |
| --- | --- | --- | --- | --- | --- | --- | --- | --- | --- | --- | --- | --- | --- | --- | --- | --- |
|  | | | **Sum of Squares** | | | | **df** | | **Mean Square** | | | | **F** | | **p** | |
| Question | |  | 8485.5 | | |  | 1 |  | 8485.5 | | |  | 45.259 |  | < .001 |  |
| Question x Condition | |  | 934.8 | | |  | 1 |  | 934.8 | | |  | 4.986 |  | 0.027 |  |
| Question x Order | |  | 1142.4 | | |  | 3 |  | 380.8 | | |  | 2.031 |  | 0.112 |  |
| Question x Condition x Order | |  | 401.5 | | |  | 3 |  | 133.8 | | |  | 0.714 |  | 0.545 |  |
| Residual | |  | 26623.3 | | |  | 142 |  | 187.5 | | |  |  |  |  |  |
|  | | | | | | | | | | | | | | | | |
| *Note.*  Type III Sum of Squares | | | | | | | | | | | | | | | | |
| **Between Subjects Effects** | | | | | | | | | | | | | |  |  |  |
|  | | **Sum of Squares** | | **df** | | **Mean Square** | | | | **F** | | **p** | |  |  |  |
| Condition |  | 5.048 |  | 1 |  | 5.048 | | |  | 0.015 |  | 0.903 |  |  |  |  |
| Order |  | 1210.034 |  | 3 |  | 403.345 | | |  | 1.200 |  | 0.312 |  |  |  |  |
| Condition x Order |  | 568.176 |  | 3 |  | 189.392 | | |  | 0.564 |  | 0.640 |  |  |  |  |
| Residual |  | 47713.398 |  | 142 |  | 336.010 | | |  |  |  |  |  |  |  |  |
|  | | | | | | | | | | | | | |  |  |  |
| *Note.*  Type III Sum of Squares | | | | | | | | | | | | | |  |  |  |
